# The Relationship between Emotional Content and Word Processing in Normal Persian Speaking Children

**Published:** 2018

**Authors:** Sousan SALEHI, Ahmad Reza KHATOONABADI, Mahmoud Reza ASHRAFI, Ghasem MOHAMMADKHANI, Saman MAROUFIZADEH, Fatemeh MAJDINASAB

**Affiliations:** 1 Department of Speech Therapy, School of Rehabilitation, Tehran University of Medical Sciences, Tehran, Iran; 2 Department of Child Neurology, Children's Medical Center, Tehran University of Medical Sciences, Tehran, Iran; 3 Department of Audiology, School of Rehabilitation, Tehran University of Medical Sciences, Tehran, Iran; 4 Department of Epidemiology and Reproductive Health, Reproductive Epidemiology Research Center, Royan Institute for Reproductive Biomedicine, ACECR, Tehran, Iran

**Keywords:** Emotional content words, Use, Comprehensibility, Familiarity, Persian

## Abstract

**Objectives:**

Emotion is a key component in language processing, but emotional words processing in children is still controversial. We aimed to investigate the relationship between emotional dimensions, arousal and valence, word familiarity, comprehension, use, and emotional content recognition. Eventually, a list of emotional content words for this age was prepared in Persian.

**Materials&Methods:**

The study was conducted in selected elementary schools in Tehran, Iran from April to June 2017. Emotional words, from adult emotional words list, were categorized into 5 groups according to their arousal and valence scores, including neutral, happy, calm, anxious and sad. Evaluation of familiarity, use, comprehension and emotional content recognition of the list was conducted with a checklist in 60 first grade children by speech and language pathologist.

**Results:**

Neutral words gained the highest score in familiarity, use, comprehension and emotional content recognition (the mean=0.74). Afterward, there were the emotional words with high valence, calm (the mean=0.64) and happy (the mean=0.52). Finally, it was found the low score for valence emotional words, sad (the mean=0.46) anxious (the mean =0.43) in end of score rating. There was a significant difference between all word groups in four aspects (*P*-value<0.001). There were no significant differences between boys and girls in four aspects.

**Conclusion:**

Neutral words are better comprehended and expressed than emotional words. Valence is more effective than arousal in emotional words. Gender was not a determinant factor in all of the aspects. An emotional word list which is comprehensible for children in Persian language was prepared.

## Introduction

Generally, emotion is defined as complicated mental state including three definite constituents: conscious experience related to psychological states, physiological response closed to nervous system reaction, and behavioral or expressive response related to motivation (1, 2). Primarily, emotions were a reflexive response to the stimulus; these responses are motions toward positive and pleasant things and away from negative and unpleasant things. Many of theories of emotion proposed “dimensional organization” for emotion (3). Accordingly, it has bipolar dimensions, including arousal and valence based on evaluative reactions (4). Valence can be defined by bipolar scales that describe constant dimension from pleasantness such as happy and pleased to unpleasantness such as unhappy and annoying (3, 5). The second dimension is arousal described by bipolar scales from an unaroused state such as calming, sleepy and relaxed to high arousal state such as excited and stimulated (3, 5). 

There is no gold standard for measuring emotion but Self-Assessment Mankin (SAM) is a widely used assessment technique that precisely measures the arousal and valence. It is a photographic nonverbal approach then it can be used in various language and culture (6).

Emotion in word can be expressed in two ways: verbal or content and non-verbal or prosodic emotion (7). Both of them have an impact on word processing (7). There is debatable data in mutual relationship between emotional aspects including arousal and valence, and emotional word processing. Emotional words processing is modulated by arousal (8, 9), for example, high arousal emotion such as fear had negative effect on information processing in children (9). Similarly, arousing words were better processed (10). On the other hand, valence is more important than arousal in word processing (11, 12). For example, an electrophysiological study indicated that valence and concreteness of word had a key role in emotional words processing (12). Negative emotional words were processed slower than positive and words with high valence slower than low valence, so valence was more important than arousal (13). Therefore, it is still controversial that which dimension of emotion (valence or arousal) is more effective in word processing. This gap in the research will be considered to find out which one is more important in word processing.

Word processing can be assessed by comprehension and expression of words. There are various factors such as frequency of word, age of acquisition and recently emotional content which has influence on word comprehension and expression (11, 14). Comprehension and expression of emotional content words were investigated in some languages. These studies were conducted in Dutch, English, and Chinese languages and they established a list of appropriate words for each age (14-17). Additionally, appropriate emotional and behavioral function in children required language competency (18). Language is a vital tool to express emotion and feelings (19). Children with language disorders experience more emotional and social problems than their peers (20). These language disorders have a wide range, from phonological disorders to pragmatics and limited vocabulary. Then, because of relationship between language and behavior, maybe, these can be the base of behavioral difficulties (21). There is no study to explore emotional content word processing in Persian speaking children and we have no emotional content words list for children. This list can be useful for language therapy in children.

The main aim of present study was to explore the relationship between emotional dimensions (arousal and valence) and word processing (familiarity, use, comprehension and emotional content recognition of words) words in Persian- speaking children 7-8 yr old. Any difference between boy and girls abilities was considered. 

## Materials & Methods

The present study had cross-sectional non–experimental research design, including 4 parts as follows:

**Preparation of tools**

This was a non-experimental descriptive cross-sectional study. Previously, 320 words of the Persian emotional words list were rated according to valence and arousal aspects by 1-7 Self-Assessment-Mankin (SAM) scale by 1200 normal Persian- speaking adult using computer. These words were selected from valid Persian dictionaries based on their frequency and use in daily living (16). As mentioned earlier, SAM is a pictorial measurement of emotion. This approach was developed for assessment of emotional state which used nonverbal graphical representations for feelings. This method has a high correlation with other verbal semantic scales (6).

**Fig 1:**
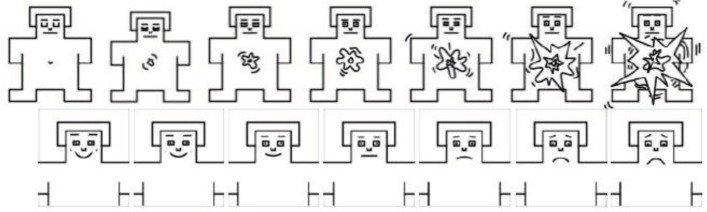
1-7 SAM scale (19) for arousal (top) and valence (down) (19).

This technique has a series of pictures which varied in arousal from sleepy calm to arousing state and valence (pleasant) from happy to sad face (Figure 1). It can be done in both computer and pencil-paper ways. Since it is a nonverbal graphical oriented scoring system, it does not depend on language ability. Then, it can be employed with non-English speaking people. However, its validity and reliability should be investigated (6). This instrument became valid and reliable in Iran, and test-retest reliability coefficient was in the range of 0.55-0.78 and concurrent validity ranged from 0.56 to 0.87 for SAM (22). We utilized this emotional word list as resource for our investigation.

Emotional words can be categorized by their scores in valence and arousal, into five groups, namely, happy, calm, sad, anxious, and neutral words (16), including:

1. Happy words including words which had scored more than 4 in both of arousal and valence.
2. Calm words consisted of words with score less than 4±S.D. in arousal and more than 4 in valence.
3. Sad words contained words which had score less than 4±S.D. in both of arousal and valence.
4. Anxious words including words which had scored more than 4±S.D. in arousal and less than 4 in valence, and finally,
5. Neutral words with score 4±S.D. in both of arousal and valence.

We categorized the words by their instruction as explained above in a five scoring and prepared the list which contained 40 happy, 78 calm, 28 sad, 80 anxious and 94 neutral words. These words were assessed in four selected dimensions of word processing, namely, familiarity, comprehension, use and emotional content recognition by a checklist which obtained content validity according to six expert speech and language pathologists and one linguist. The checklist was administered by one experienced speech and language pathologist in children for each word and it has yes/no scoring system, 1 for yes and 0 for no. The used question is following:

1. “Familiarity” was evaluated by asking: Is this word heard or not? Yes/no

2. “Comprehension” was evaluated by word definition: Is the definition clear? Yes/no

3. “Use/Expression” was assessed by making a sentence with the words. Is the sentence meaningful? Yes/no

4. Before emotional words were categorized into three main groups based on their scores in arousal and valence, including neutral, positive (calm and happy) and negative (sad and anxious). To describe this categorization to children, we used good, bad and moderate (not good, not bad) for positive, negative and neutral categories, respectively. For example, “family” is a calm category then it is described as a good word for children. Then, for “Recognition of emotional content” evaluation, Child was asking: is this word good, bad or moderate? (If the subject recognize emotional content of words inconsistent with emotional categorization/yes, otherwise/no).

For example, is “family” a good, bad or moderate word? If the child says: good, it is correct because it was a positive word. 

**Participants**

Participants were 60 (30 boys and 30 girls) normal Persian-speaking children, their age ranged from 79-89 months (S.D. =3.46). They were recruited at random from first-grade classes in two public elementary schools from April to June 2017. These schools including a girl’s school and a boy’s school were selected randomly from central areas in Tehran, Iran which are commonly moderate area. The files of all first-grade students in each school were checked and the names of students who met inclusion criteria were written down. Then, our participants were selected randomly from these written names.

Inclusion criteria of participants were: 1) native monolingual Persian speaking children; 2) middle level of socio-economic status of families based on their family information in their files and teacher’s opinion; 3) No history of psychiatric and neurological disorders based on their medical history documents in schools, 4) No reading or other speech and language disorders, according to speech and language pathologist assessment. 

All parents included children gave the parental informed consent form. This study has been approved by the Research Council, School of Rehabilitation, Tehran University of Medical Sciences.

**Procedure**

Four questions in the checklist were asked for 320 words in emotional word list and the score was recorded by 1-0 scoring system. It takes almost 1.5 h for each child. There was a timeout for probable tiredness and finally the children have received a gift for their cooperation. All participants were evaluated in one private quiet classroom individually by one experienced speech and language pathologist. 

One expert speech and language pathologist asked the questions for familiarity, comprehension, use, and recognition of emotional content and record the child’ response. Their scores were calculated for each word based on their verbal recorded responses. Total score for each word was between 0-4. Questions and response are explained in Table 1.

**Table 1:** Procedure of the checklist scoring

	SLP’ question	Child’s tasks	Scoring base	Scores
Familiarity	Is this word (chair) heard or not?	Saying Yes / no	Yes or no	Yes =1 No =0
Comprehension	What is the meaning of this word (chair)?	Defining the word, for example: the thing which sitting on it Or using synonyms	Is the definition clear? Yes/no Is the synonym correct? Yes/ no	Yes =1 No =0
Use / expression	Can you make a sentence with this word (chair)?	Making a meaningful sentence with this word. We have a chair in the room	Is the sentence making sense or not? Yes/no	Yes =1 No =0
Recognition of emotional content	Is this word (chair) good/bad or moderate?	Deciding the word is good or bad or moderate. Chair is a neutral word then correct response is moderate.	Good for happy and calm words is yes Bad for sad and anxious words is yes Moderate for neutral words is yes Otherwise No	Yes =1 No =0

**Statistical Analysis**

All statistical analyses were performed using IBM SPSS Statistics for Windows, ver. 22.0 (IBM Crop., Armonk, NY, USA). The Shapiro-Wilk test was used to evaluate the normality of the data. A Kruskal-Wallis test, followed by the Dunn posthoc test, was used to compare between categories of words. Relationships between dependents variables including familiarity, comprehension, use, emotion recognition and mean of scores were examined by Spearman correlation coefficient. All statistical tests were two-sided and a *P*-value<0.05 was considered statistically significant.

## Results

The subjects were 60 first grade school children, their age ranged from 79-89 months (S.D. =3.46). They also were matched in gender and socioeconomic status. 

Strong positive correlations were found between all dependent variables including, familiarity, use, comprehension and emotional content recognition (ranging from 0.91 to 0.98) (Table 2).

**Table 2:** Means, standard deviations, and correlations among study variables

	**Mean (SD)**	**Familiarity**	**Comprehension**	**Use**	**Emotional Content Recognition**	**Mean of score**
**1. Familiarity**	0.67 (0.38)	1				
**2. Comprehension **	0.43 (0.29)	0.930^a^	1			
**3. Use/Expression **	0.62 (0.39)	0.980	0.934	1		
**4. Emotional Content Recognition **	0.63 (0.38)	0.966	0.910	0.959	1	
**5. Mean of score**	0.59 (0.36)	0.974	0.973	0.975	0.971	1

SD: standard deviation

a All correlations are significant at 0.001 level (*P*<0.001)

All word processing variables had strong relationship. It means these variables for word processing had been selected appropriately. The strongest relationship was between use and familiarity (0.98).

Scores in different dependent variables for five categories of words are exhibited in Table 3. 

**Table 3:** Values of five emotional categories

	**Calm**	**Anxious**	**Neutral**	**Happy**	**Sad**	***P*** **-Value** †
**Familiarity**	0.73 (0.36)^a^^b^	0.51 (0.38)^c^	0.82 (0.31)^a^	0.61 (0.40)^b^^c^	0.55 (0.43)^b^^c^	<0.001
**Comprehension **	0.48 (0.28)^a^	0.32 (0.28)^b^	0.58 (0.26)^a^	0.32 (0.26)^b^	0.29 (0.26)^b^	<0.001
**Use **	0.66 (0.37)^b^	0.45 (0.38)^c^	0.79 (0.33)^a^	0.57 (0.40)^b^^c^	0.50 (0.42)^b^^c^	<0.001
**Emotional content Recognition **	0.70 (0.35)^a^^b^	0.46 (0.36)^b^	0.78 (0.32)^a^	0.59 (0.39)^a^^b^	0.49 (0.40)^b^^c^	<0.001
**Mean of scores**	0.64 (0.33)^a^^b^	0.43 (0.35)^c^	0.74 (0.30)^a^	0.52 (0.36)^b^^c^	0.46 (0.37)^b^^c^	<0.001

Values are given as mean (SD)

† Kruskal-Wallis test followed by the Dunn post-hoc test

a-c Groups followed by the same letter are not significantly different at the 0.05 level.

**Appendix 1:** Children emotional words list

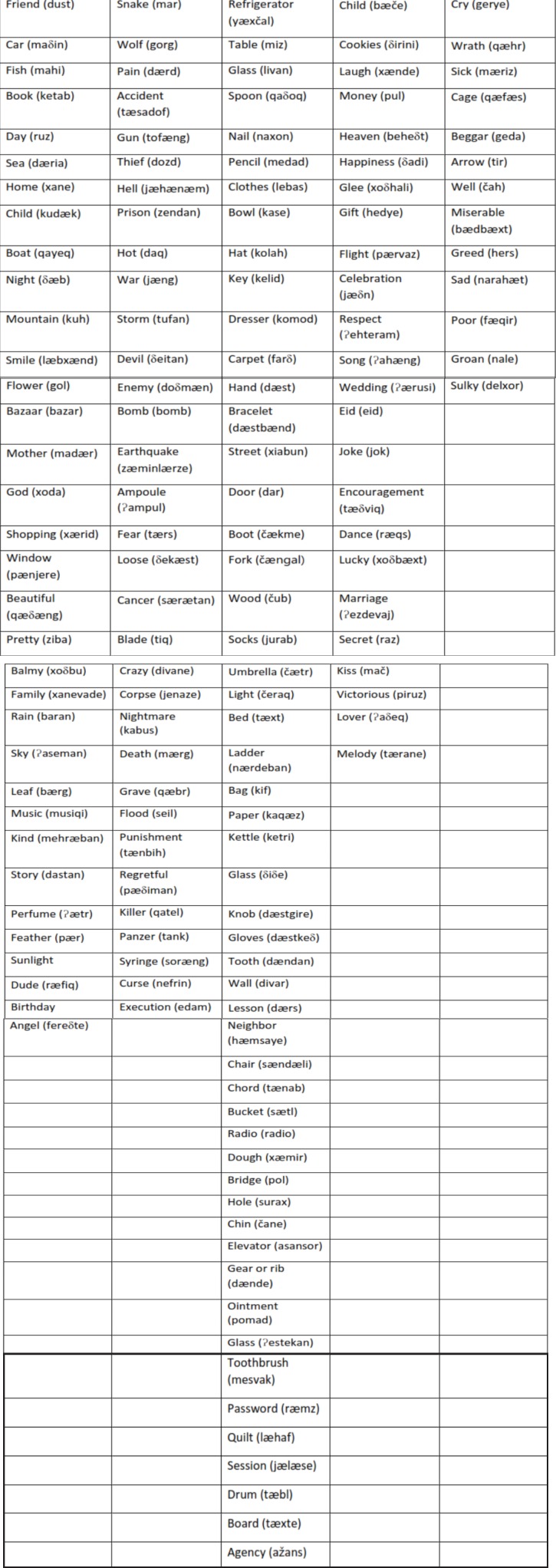

**Familiarity:** Neutral words were the most familiar for children (mean =0.82). Calm words were the next group (mean=0.73), happy words after calm words (mean=0.61). Sad and anxious words did not have marked differences, by mean 0.55 and 0.51, respectively. Generally, the words with high valence were more familiar than low valence, after neutral words. Differences between all groups were significant (*P*-value < 0.001).

**Comprehension: **Likewise familiarity, neutral words had the most score in comprehension (mean=0.58) which followed by calm words (mean=0.48). Anxious and happy words were at the same level (mean=0.32). Finally, sad words had the lowest score (mean=0.29). Then neutral and high valence words had more score in comprehension variable. There was significant difference between all word groups (P-value < 0.001).

**Use**: In the case of use, neutral words had been taken as the most score (mean=0.79). Then, calm (mean=0.66) and happy words (mean=0.57) were the next. These words were followed by sad words (mean=0.5) and anxious words (mean=0.45) had the least score of use. Difference between mean score in all word groups was significant (*P-*value < 0.001).

**Emotional content recognition**: There was the same pattern in emotion recognition variable, first there were neutral words (mean=0.78), next calm (mean=0.7) and happy (mean=59) and following by sad (mean=0.49) and finally, anxious words (mean=0.46). As with other variables, significant difference was in all word groups (P-value < 0.001).

**Mean of scores**: Overall, mean of scores in neutral words was the most (mean=0.74), next calm (mean=0.64) and happy (mean=0.52) respectively, and then anxious (mean=0.43) and sad words (mean=0.46). In other words, the words without emotional content (neutral words) had the highest mean of score, and then the words with high valence were followed by the words with low valence. Altogether, differences in all word groups were statistically significant (*P*<0.001). 

Although there were no significant differences between girls and boys in mean of scores in all word groups (*P*>0.05), some words in each word category had significant difference between boys and girls. These words included: lover (mӕδuq), life (zendegi), waterfall (ʔabδar) and calm (ʔaram) in clam category, in these words boys had more scores. In anxious category, girls had more score in curse (nefrin), disaster (mosibӕt) and regretful (pӕδiman) and boys had more scores in cartridge (feδӕng), bandit (rahzӕn), terrorist (terorist), panzer (tank). Considering neutral category, boys were better in ladder (nærdeban), toothbrush (mesvak), steel (fulad), gear or rib (dӕnde), soil (xak), asphalt (ʔasfalt), and iron (ʔahӕn) words. Boys also were significantly better in flight (pӕrvaz), song (ʔavaz), gift (hedie) and happy (xoδhal) in happy words and cage (qӕfӕs) in sad words (*P*-value<0.05). 

Conclusively, the words with mean of scores below 0.5 including 138 words in all word groups were omitted from the list, and finally, the children emotional content word list with 182 words was prepared. 

## Discussion

We aimed to investigate the relationship between emotional content and some aspects of words processing including use, comprehension, familiarity and emotional content recognition of words, by considering two dimensions of emotion including valence and arousal. 

Neutral words got the highest score in all dimensions of word processing. As well as, calm and happy words got higher score than sad and anxious. Pleasant had stronger relationship than arousal and furthermore, emotional words with high pleasant were processed more highly accurate than others. 

We found that neutral words were the best across other categories in all aspects of word processing, including use, comprehension, familiarity, and emotional content recognition. By contrast, emotional content assists word processing (11). This difference can be explained by the type of these words. In the present study by using Persian emotional words list (16), all neutral words were nouns, thus those appear earlier in development. Additionally, concreteness and frequency are effective factors in word processing (12), it is valid in this word category (23), the neutral words which are nouns are high frequent and concrete/ subjective in compare to emotional words which are usually adjective/objective. Accordingly, it can be reasons why neutral words have the highest scores in our investigation. While valence and arousal are two main dimensions of emotion, just valence is taken to account for word processing based on our analysis. Valence is likely an effectual factor in comprehension and use of words and emotional content recognition. Apparently, it is in contrast to study which declares emotion is adjusted by the arousal of the emotional words (8). Our results are consistent of the results of other studies which argued the valence of the emotional words was more important than arousal in word processing (11, 12). Pleasure (valence) has an important act in word processing in Persian speaking children rather than arousal. In other words, pleasantness facilitated emotional content processing. 

Therefore, pleasure (valence) is important in language processing then it could be considered in speech and language assessment and treatment procedures in Persian language.

There was no significant difference between boys' and girls' performance, in consistent with another study (24). However, there is only one study contrary to our findings suggested that girls produced more emotional content words than boys (17). Generally, emotional content word processing is not affected by gender, but we have some girlish and boyish words that mentioned in results.

Additionally, there are researches in developing comprehensible emotion words list for children in other languages (14), at the end of analysis, we prepared a list of emotional words which is suitable for 7-yr-old children (Appendix 1). Since there is not emotional words list in Persian language for children, the list can be a preliminary list for next researches.

The limitation in this study was that the most of neutral words are nouns and the most of emotional content words are adjectives in Persian language. Even if nouns and adjectives were matched in frequency and length, these had different type of word and age of acquisition. Emotional content word processing investigation can be conducted by electrophysiological methods, like Event-Related Potentials which has high temporal resolution and it can illustrate underlying neural mechanism in arousing and pleasant word processing.

**In conclusion,** neutral words are processing better than emotional words. Valence is more effective than arousal in emotional aspect, in the other words, pleasant words processing is better than other emotional words. 
